# Initiation of Antiretroviral Therapy (ART) at Different Stages of HIV-1 Disease Is Not Associated with the Proportion of Exhausted CD8^+^ T Cells

**DOI:** 10.1371/journal.pone.0139573

**Published:** 2015-10-01

**Authors:** Sanne Skov Jensen, Anders Fomsgaard, Tine Kochendorf Larsen, Jeanette Linnea Tingstedt, Jan Gerstoft, Gitte Kronborg, Court Pedersen, Ingrid Karlsson

**Affiliations:** 1 Virus Research & Development Laboratory, Department of Microbial Diagnostic and Virology, Statens Serum Institut, Copenhagen, Denmark; 2 Department of Infectious Diseases, Odense University Hospital, Odense, Denmark; 3 Infectious Disease Research Unit, Clinical Institute, University of Southern Denmark, Odense, Denmark; 4 Viro-immunology Research Unit, Department of Infectious Diseases, Copenhagen University Hospital, Copenhagen, Denmark; 5 Department of Infectious Diseases, Copenhagen University Hospital, Hvidovre, Denmark; Karolinska Institutet, SWEDEN

## Abstract

CD8^+^ T cell-restricted immunity is important in the control of HIV-1 infection, but continued immune activation results in CD8^+^ T cell dysfunction. Early initiation of antiretroviral treatment (ART) and the duration of ART have been associated with immune reconstitution. Here, we evaluated whether restoration of CD8^+^ T cell function in HIV-1-infected individuals was dependent on early initiation of ART. HIV-specific CD107a, IFNγ, IL-2, TNFα and MIP-1β expression by CD8^+^ T cells and the frequency of CD8^+^ T cells expressing PD-1, 2B4 and CD160 were measured by flow cytometry. The frequency of CD8^+^ T cells expressing the inhibitory markers PD-1, 2B4 and CD160 was lower in ART-treated individuals compared with ART-naïve individuals and similar to the frequency in HIV-uninfected controls. The expression of the three markers was similarly independent of when therapy was initiated. Individuals treated before seroconversion displayed an HIV-specific CD8^+^ T cell response that included all five functional markers; this was not observed in individuals treated after seroconversion or in ART-naïve individuals. In summary, ART appears to restore the total CD8^+^ T cell population to a less exhausted phenotype, independent of the time point of initiation. However, to preserve multifunctional, HIV-1-specific CD8^+^ T cells, ART might have to be initiated before seroconversion.

## Introduction

CD8^+^ T cells play a well-documented role in clearing and/or controlling viral infections [1]. The reduction in viremia when virus-specific T cell-mediated immunity emerges [2], the necessity of CD8^+^ T cells in the control of simian immunodeficiency virus (SIV) in a macaque model [3] and the loss of immune control by viral escape mutations [4] all show the importance of CD8^+^ T cell-restricted immunity in the control of HIV-1 infection.

Chronic HIV-1 infection results in CD8^+^ T cell dysfunction [5]. Several of the CD8^+^ T cell functions are lost early during infection, e.g., the ability to secrete IL-2 and to proliferate as well as cytotoxic function. However, the ability to secrete IFNγ persists for a longer time [5]. When the viral load is high and help from the CD4^+^ T cells is poor, virus-specific effector CD8^+^ T cells lacking effector function appear [5–7]. Expression of inhibitory markers such as PD-1, 2B4 and CD160 has been shown to be increased on CD8^+^ T cells during chronic infection [8–11] and to be decreased by the introduction of ART in HIV-infected individuals [11]. Expression of PD-1 has been linked to less proliferative capacity in CD8^+^ T cells. In addition, co-expression of PD-1, 2B4 and CD160 is associated with an exhausted phenotype; impaired proliferation; and a reduced capacity to produce IFNγ, perforin and IL-2 [12, 13].

Previous studies in macaques demonstrated better long-term control of SIV replication after treatment was withdrawn if ART was administered early in the infection [14, 15]. Comprehensive studies in HIV-1-infected individuals receiving ART within the first four months of infection have demonstrated an enhanced likelihood of recovery of CD4^+^ T cell counts [16]. In addition, ART interruption in HIV-1-positive individuals who were treated at the time of primary infection showed evidence of long-term immunological control [17–19]. Moreover, ART initiated in individuals positive for HIV-1 RNA but negative for p24 antigen and anti-HIV antibodies prevented loss of Th17 cell numbers and function compared with ART in seroconverted individuals. For seroconverters, the Th17 cell numbers, but not their functionality, were restored [20]. Prolonged ART initiated at the time of HIV-1 seroconversion is associated with immunovirological features that resemble those of long-term non-progressors [21]. Collectively, these findings demonstrate that the timing of ART initiation and the duration of treatment are crucial for immune reconstitution.

We wanted to evaluate whether the time of treatment initiation influenced the restoration of CD8^+^ T function during HIV-1 infection. It appeared that long-term ART (here defined as >2 years) was sufficient to normalize the expression of the inhibitory markers PD-1, 2B4 and CD160 to the levels observed in HIV-negative individuals, irrespective of the time of ART initiation. However, initiating therapy early, before seroconversion, might preserve the multifunctional, HIV-1-specific CD8^+^ T cell response.

## Study Participants and Methods

### Ethics statement

The study was approved by the National Committee for Health Research Ethics of the Danish Ministry of Health (H-3-2011-031 and H-3-2012-104). All study participants provided written informed consent.

### Study participants

A total of 49 HIV-1-positive individuals followed at the University Hospitals of Copenhagen, Rigshospitalet and Hvidovre or at Odense University Hospital in Denmark were recruited into the study. Eight individuals were treated before seroconversion, seroconversion is defined as positive for RNA and/or p24 antigen but not for antibody, i.e. treatment was initiated before Fiebig phase III. 11 individuals started ART with a CD4^+^ T cell count >350 cells/μl blood; 19 individuals had started ART with a CD4^+^ T cell count <350 cells/μl blood; and 11 individuals were ART naïve. Moreover, 11 HIV-uninfected individuals were recruited into the study. Participants on ART were viral suppressed for at least two years before sampling. Whole blood or peripheral blood mononuclear cells (PBMCs) were used for analysis. The PBMCs were obtained by density-gradient centrifugation and were cryopreserved until the analysis was performed. Table 1 outlines the clinical characteristics of the study participants.

**Table 1:** Clinical characteristics of study participants.

Group	Study ID	Sex	Age, years	Duration of ART, years	Treatment at sampling time	CD4^+^ T cells count at ART initiation, cells/μl	CD4^+^ T cell count, cells/μl	HIV-1 plasma viral load, copies/ml	HIV-1 subtype
ART before seroconversion, n = 8	3	m	69	8	Truvada, Norvir, Prezista	460	940	19	B
	7	m	40	5	Kivexa, Stocrin	238	696	<20	B
	8	m	30	5	Epivir, Viread, Viramune	570	1500	19	B
	13	m	29	2	Atripla	520	950	79	B
	21	m	45	6	Atripla	320	1100	19	B
	22	m	35	5	Kivexa, Norvir, Reyataz	530	900	19	B
	65	m	58	9	Atripla	412	994	<20	B
	66	m	38	5	Atripla	490	521	<20	B
Median (IQR)	-	-	39 (31–55)	5 (5–7)	-	475 (343–528)	945 (747–1074)	20(19–20)	-
ART at CD4^+^ T cell count >350 cells/μl, n = 11	2	m	45	5	Atripla	567	925	39	B
	4	m	41	2	Viramune, Truvada	510	640	19	B
	18	m	37	3	Truvada, Isentress	520	530	19	B
	25	m	28	6	Kivexa, Stocrin, Sustiva	500	680	20	B
	27	m	39	4	Atripla	350	710	19	B
	28	m	41	4	Truvada, Viramune	390	660	19	B
	72	m	47	3	Truvada	672	867	<20	B
	73	m	64	5	Truvada, Prezista, Norvir	418	269	53	B
	80	m	43	2	Atripla	492	1170	NA	B
	83	m	45	2	Atripla	492	575	20	A
	122	m	66	16	Tivicay, Truvada	360	670	<20	NA
Median (IQR)	-	-	43 (39–47)	4 (2–5)	-	492 (390–520)	670 (575–867)	20(19–25)	-
ART at CD4^+^ 9T cell count <350 cells/μl, n = 19	5	m	39	6	Atripla	160	510	<20	B
	15	f	24	2	Truvada, Norvir, Reyataz	330	890	19	D
	16	m	58	3	Atripla	200	560	19	B
	17	m	40	5	Epivir, Viread, Stocrin, Sustiva	180	520	39	B
	31	m	41	4	Truvada, Viramune	150	750	19	B
	32	f	65	7	Epivir, Norvir, Prezista	250	360	19	C
	35	m	44	4	Truvada, Isentress	310	520	19	B
	55	m	41	6	Atripla	52	272	<20	B
	62	m	50	5	Atripla	40	513	20	B
	89	m	30	10	Atripla	190	757	<20	B
	90	m	51	8	Atripla	105	885	<20	B
	92	m	38	5	Norvir, Prezista, Viread, Epivir	157	1240	28	B
	94	m	69	8	Atripla	197	565	<20	B
	96	f	32	8	Atripla	188	446	912	G
	97	m	59	4	Atripla	193	756	20	B
	98	f	47	5	Atripla	199	504	20	B
	118	f	67	17	Norvir, Reyataz, Ziagen, Viread, Viramune	50	570	<20	B
	119	m	68	16	Truvada, Isentress	260	610	<20	NA
	120	m	59	18	Kivexa, Tivicay	180	670	<20	NA
Median (IQR)	-	-	47 (39–59)	6 (4–8)	-	188 (150–200)	565 (510–756)	20(19–20)	-
ART-naïve, n = 11	9	m	43	-	-	-	620	5,687	B
	10	m	46	-	-	-	530	414,911	AE
	36	m	34	-	-	-	570	7,737	B
	38	m	36	-	-	-	1100	1,600	B
	41	m	48	-	-	-	1200	40,996	B
	45	m	42	-	-	-	710	231	NA
	48	m	49	-	-	-	1100	11,747	B
	101	f	44	-	-	-	749	1,140	NA
	113	f	54	-	-	-	862	298	NA
	121	m	43	-	-	-	640	29,243	B
	123	m	57	-	-	-	500	33,001	NA
Median (IQR)	-	-	44 (42–49)		-	-	710 (570–1100)	7737 (1140–33001)	-

f: Female; m: Male; NA; Not avaliable; Interquartile Range (IQR)

### Clinical parameters for study participants

The plasma viral loads were quantified using the COBAS AmpliPrep/COBAS TaqMan HIV-1 Test, version 2.0 (Roche Diagnostics, Copenhagen, Denmark). The absolute CD4^+^ T cell counts were determined using the FACSCount system (BD Biosciences, San Jose, CA) according to the manufacturer’s protocol.

### Staining of markers on CD8^+^ T cells

Whole blood or PBMCs were stained. The difference in marker expression between CD8^+^ T cells in whole blood and thawed PBMCs was first established. We found differences in the expression levels of all markers examined (CD45RO, CCR7, CD27, 2B4, PD-1 and CD160), which made it impossible for us to compare and use the results from stained PBMCs and whole blood (data not shown). For this reason, we only report marker expression analyses performed on whole blood from 44 individuals: 4 treated before seroconversion, 6 treated at a CD4^+^ T cell count >350 cells/μl, 13 treated at a CD4^+^ T cell count <350 cells/μl, 10 ART-naïve individuals and 11 HIV-uninfected controls. Due to the limited number of study participants assessed in the phenotype analysis, the individuals treated before seroconversion and the individuals treated at a CD4^+^ T cell count >350 cells/μl were analyzed as one group. For the analysis, 100 μl of whole blood was incubated for 20 minutes at room temperature in the dark in the presence of LIVE/DEAD® Fixable Dead Cell Stain (Life Technologies, Carlsbad, CA, USA), anti-CD3 PerCP (BD Biosciences), anti-CD8 Qdot 605 (Life Technologies), anti-2B4 FITC (BioLegend, London, United Kingdom), anti-CD45RO APC-H7 (BD Biosciences), anti-PD-1 PECy7 (eBioscience, San Diego, CA), anti-CD27 Alexa Fluor 700 (eBioscience), anti-CCR7 PerCP/Cy5.5 (BioLegend) and anti-CD160 PE (BioLegend) antibodies.

The antibodies were pre-titrated to determine the optimal concentration for staining. After incubation with the antibodies, the whole blood was lysed for 20 minutes at room temperature using BD FACS Lysing Solution (BD Biosciences). The cells were then washed and fixed using BD Stabilizing Fixative (BD Biosciences). The fluorescence levels on the stained cells were measured using a BD LSR II and analyzed using FlowJo version 8.8.7 (Tree Star, Ashland, OR). For detailed gating strategies, see S1 Fig. In order to define cells positive for a certain marker, the corresponding IgG isotype controls were used. The surface markers CD27, CD45RO, and CCR7 were used to define memory cell populations. We defined central memory (CM) cells as CD45RO^+^CD27^+^CCR7^+^, transitional memory (TM) cells as CD45RO^+^CD27^+^CCR7^-^, effector memory (EM) cells as CD45RO^+^CD27^-^CCR7^-^, effectors as CD45RO^-^CD27^-^CCR7^-^ and naïve CD8^+^ T cells as CD45RO^-^CD27^+^CCR7^+^. Naïve CD8^+^ T cells, the population with the lowest levels of the inhibitory markers, were used as a reference to define cells with higher expression levels of PD-1, 2B4 and CD160.

### Intracellular cytokine staining assay

The number of cells accessible limited the intracellular cytokine staining assay to being performed on cells from 34 HIV-1-infected individuals who were tested for CD107a and an intracellular cytokine/chemokine response, as described in a previous study [22]. Eight individuals were treated before seroconversion, 5 were treated at a CD4^+^ T cell count >350 cells/μl, 14 were treated at a CD4^+^ T cell count <350 cells/μl, and 7 were ART-naïve individuals. ART-naïve individuals with a viral load <2000 copies/ml were excluded from the intracellular cytokine analysis to avoid bias due to controllers. In brief, CD107a and intracellular IFNγ, IL-2, TNFα and MIP-1β were analyzed by stimulating 0.5x10^6^ PBMCs/ml with HIV-antigen peptide pools (8 μg/ml for each peptide) for 6 hours at 37°C and 5% CO_2_. The peptide pools contained overlapping (by 11 amino acids) 15-mers covering the full-length HIV-1 consensus sequence (subtype B; National Institutes of Health AIDS Research and Reference Reagent Program). A total of 757 peptides were divided into 5 pools (Gag, Pol, Env, Nef and TRVVV). Staphylococcus enterotoxin B (SEB; Sigma-Aldrich, St. Louis, MO) (1 μg/ml) was used as stimulation in the positive control, whereas non-stimulated samples served as negative controls. A control for virus specificity was also used, consisting of 15-mers of human cytomegalovirus (HCMV) peptides (8 μg/ml for each peptide) overlapping by 11 amino acids (National Institutes of Health AIDS Research and Reference Reagent Program). Brefeldin-A (12.5 μg/ml) and anti-CD107a PE (BD Biosciences) were added at the beginning of the stimulation. After stimulation, the cells were incubated with EDTA and then stained for 20 minutes using a LIVE/DEAD® Fixable Dead Cell Stain Kit (Life Technologies), followed by 10 minutes of permeabilization using the Cytofix/Cytoperm Kit (BD Biosciences) according to protocol. Finally, the cells were stained in the dark with anti-CD3 PerCP (BD Biosciences), anti-CD4 Qdot 605 (Invitrogen), anti-CD8 APC-H7 (BD Biosciences), anti-MIP-1β FITC (R&D Systems, Minneapolis, MN), anti-IL-2 APC (BD Biosciences), anti-TNFα Alexa Fluor 700 (BD Biosciences) and anti-IFNγ PE-Cy7 (BD Biosciences). The fluorescence levels on the stained cells were measured using a BD LSR II and analyzed using FlowJo version 8.8.7 (Tree Star, Ashland, OR). For detailed gating strategies, see S2 Fig. Samples were considered positive if the following criteria were met: the percentage of CD107a- or cytokine-positive cells was greater than 2 times background (non-stimulated cells), the percentage of CD107a- or cytokine-positive cells was greater than 0.05% after background subtraction, and the population of positive cells was larger than 10 cells [22]. For FACS plots representing HIV-specific responses from individuals in each group see S3 Fig, S4 Fig, S5 Fig and S6 Fig.

### Statistical analysis

The data analysis was performed using GraphPad Prism 6 software version 6.0c (GraphPad Software Inc., La Jolla, CA), and the medians and interquartile ranges are illustrated in Table 1 and the figures. All data were tested for normality. The differences between the groups were tested using non-parametric one-way ANOVA (Dunn’s multiple comparisons test and the Kruskal-Wallis test) or parametric one-way ANOVA (Tukey’s multiple comparisons test). Polyfunctional data were analyzed in PESTLE version 1.7 and SPICE version 5.35 (provided by Mario Roederer, ImmunoTechnology Section, Vaccine Research Center/National Institute of Allergy and Infectious Diseases/National Institutes of Health, MD).

## Results

### The frequency of CD8^+^ T cells expressing inhibitory markers was lower in ART-treated individuals compared with untreated individuals, independent of the time of therapy initiation

**Fig 1:**
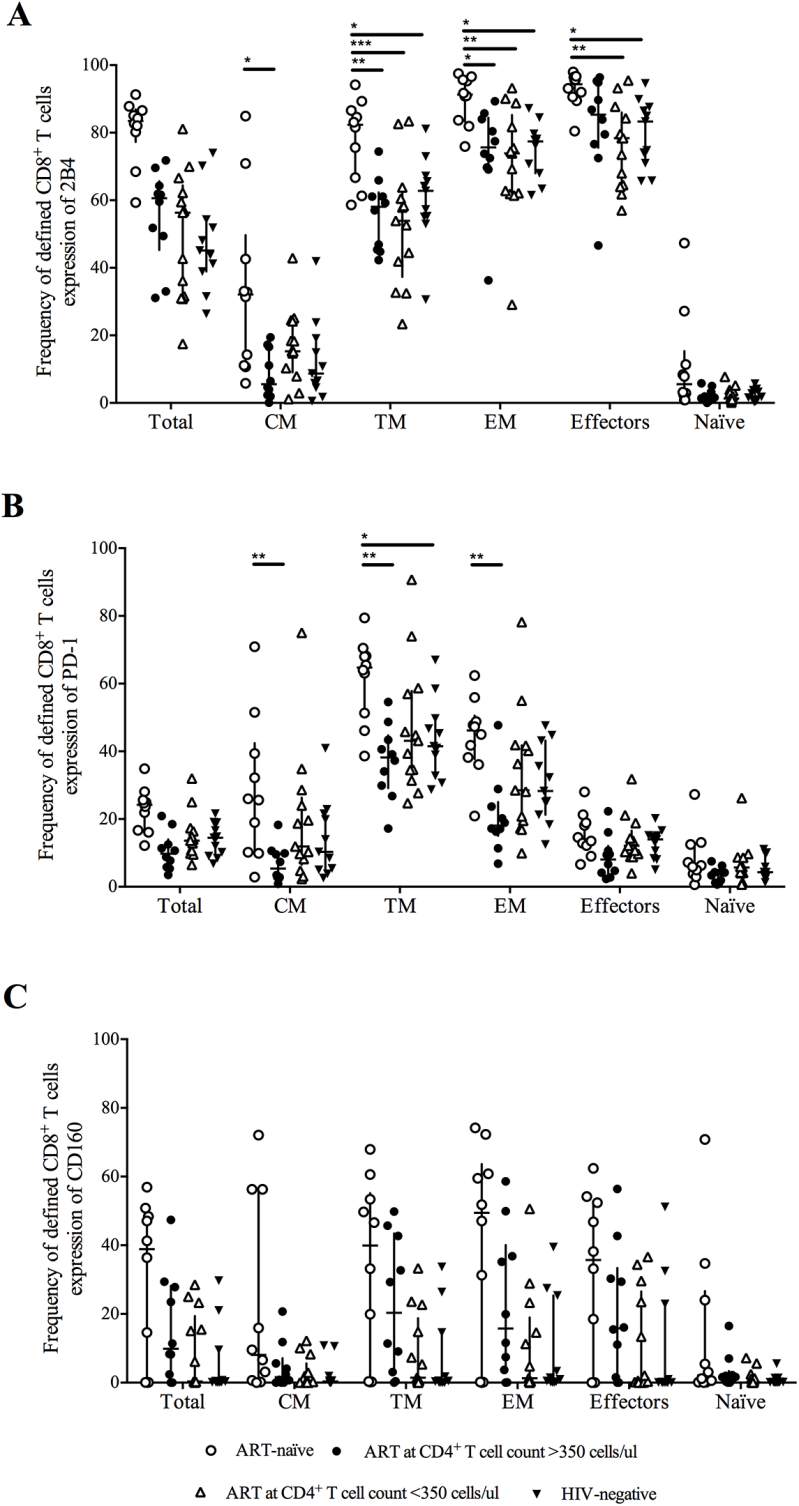
2B4 and PD-1 in individuals on ART normalizes to the levels observed for HIV-negative controls. Frequencies of total and memory CD8^+^ T cells expressing the inhibitory receptors 2B4, PD-1 and CD160. A) The frequency of CD8^+^ T cells expressing 2B4 was significantly higher in ART-naïve individuals compared with ART-treated individuals and the HIV-negative controls, independent of when ART was initiated. B) The frequency of CD8^+^ T cells expressing PD-1 was significantly higher in ART-naïve individuals compared with ART-treated individuals and the HIV-negative controls, independent of when ART was initiated. C) No differences were found in the frequency of CD8^+^ T cells expressing CD160 between ART-naïve, ART-treated and HIV-negative individuals. * means 0.01<p<0.05; ** means 0.001<p<0.01; *** means 0.0001<p<0.001.

The objective was to evaluate whether the time point when ART was initiated had an impact on the restoration of CD8^+^ T cell function during chronic HIV-1 infection. To pursue this aim, the frequencies of total and memory CD8^+^ T cells expressing the inhibitory markers 2B4, PD-1 and CD160 were examined in four groups of individuals: ART-naïve individuals, individuals with ART initiated early (at a CD4^+^ T cell count >350 cells/μl blood, including 4 individuals treated before seroconversion), individuals with ART initiated late (at a CD4^+^ T cell count <350 cells/μl blood) and HIV-negative controls (Fig 1). The frequency of CD8^+^ T cells expressing 2B4 was lower in individuals receiving ART compared with ART-naïve individuals (Fig 1A). No difference was observed depending on when ART was initiated, and the level in ART-treated individuals was similar to that in HIV-uninfected individuals (Fig 1A). The CD8^+^ T cells expressing 2B4 were mainly found within the TM, EM and effector subsets. In the CM CD8^+^ T cells, the 2B4 expression deviated between ART-naïve individuals and individuals treated at a CD4^+^ T cell count >350 cells/μl (Fig 1A). The frequencies of TM and EM CD8^+^ T cells expressing 2B4 were significantly higher in ART-naïve individuals compared with both HIV-1-positive individuals treated with ART and HIV-negative individuals. For the effector CD8^+^ T cells, the 2B4 expression was significantly higher in ART-naïve individuals compared with individuals treated at a CD4^+^ T cell count <350 cells/μl and with HIV-negative controls (Fig 1A). The expression of PD-1 was primarily found on TM and EM CD8^+^ T cells. The frequencies of CM, TM and EM CD8^+^ T cells expressing PD-1 were significantly higher in ART-naïve individuals compared with individuals treated at a CD4^+^ T cell count >350 cells/μl (Fig 1B). The frequency of TM CD8^+^ T cells expressing PD-1 also deviated between ART-naïve individuals and HIV-negative controls (Fig 1B). As for 2B4 expression, PD-1 expression during ART appeared to normalize to the levels observed for HIV-negative individuals, independent of the time of therapy initiation (Fig 1B).

No differences were found in the frequency of CD8^+^ T cells expressing CD160 (Fig 1C) between ART-naïve, ART-treated and HIV-negative individuals. The staining intensity (MFI) of the markers (2B4, PD-1 and CD160) on total, effector and memory CD8^+^ T cells was also examined, but no differences were found between ART-naïve and ART-treated individuals, even when taking the time of ART initiation into account (data not shown).

**Fig 2:**
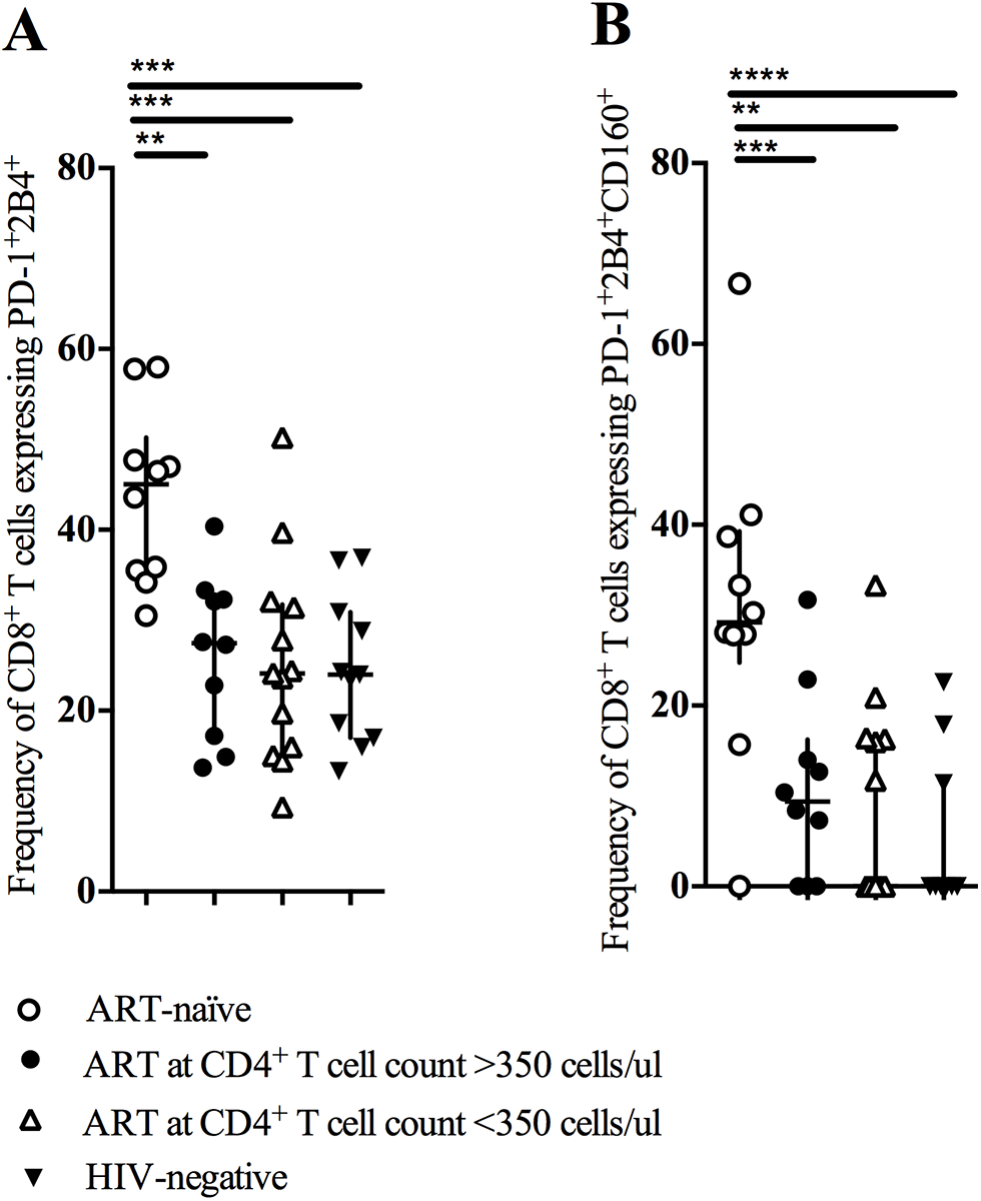
Co-expression of PD-1, 2B4 and CD160 is independent of when ART is initiated. Co-expression of PD-1 and 2B4 or of PD-1, 2B4 and CD160 was tested in ART-naïve, ART-treated and HIV-negative individuals. A) The frequency of CD8^+^ T cells co-expressing PD-1 and 2B4 was significantly higher in ART-naïve individuals compared with individuals receiving ART, independent of when ART was initiated. B) The frequency of CD8^+^ T cells co-expressing PD-1, 2B4 and CD160 was significantly higher in ART-naïve individuals compared with ART-treated individuals and HIV-negative individuals, independent of the time of treatment initiation. ** means 0.001<p<0.01; *** means 0.0001<p<0.001 and; **** means p<0.0001.

When examining the co-expression of the inhibitory markers, the difference observed between ART-naïve and ART-treated individuals was even more pronounced. The frequency of CD8^+^ T cells co-expressing PD-1 and 2B4 (Fig 2A) as well as the frequency of CD8^+^ T cells co-expressing PD-1, 2B4 and CD160 (Fig 2B) was significantly higher in ART-naïve individuals compared with individuals receiving ART, independent of when ART was initiated. CD8^+^ T cells co-expressing PD-1, 2B4 and CD160 was mainly observed for CM, TM, and EM cells, and not for effectors (data not shown). The expression of these inhibitory markers was not different between individuals receiving ART and HIV-negative individuals, suggesting that ART restores the function of CD8^+^ T cells independent of when ART was initiated.

### Individuals treated before seroconversion had a multifunctional, HIV-1-specific CD8^+^ T cell response that included expression of IL-2

**Fig 3:**
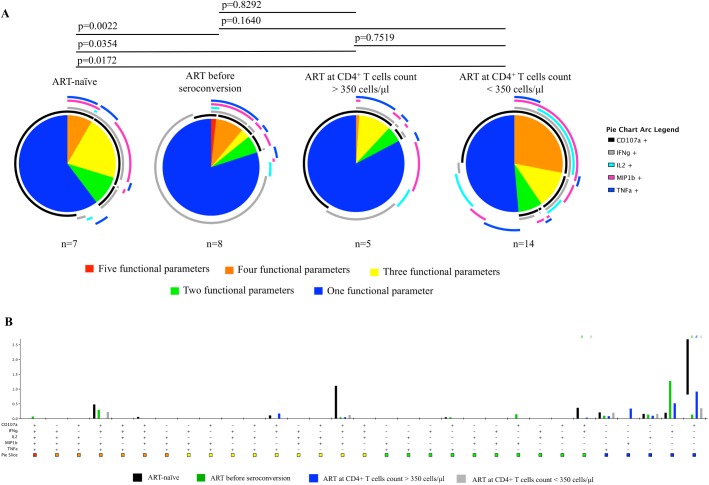
Only individuals treated before seroconversion respond with IL-2 expression in combination with CD107a, IFNγ, IL-2 and TNFα. A) The HIV-specific CD107a, cytokine (IFNγ, IL-2 and TNFα) and chemokine (MIP-1β) responses in CD8^+^ T cells were different between ART-naïve individuals and individuals treated with ART. B) ART-naïve individuals had a significantly greater CD107a response compared with treated individuals, independent of the time of treatment initiation. In addition, the co-expression of CD107a and IFNγ was significantly higher in ART-naïve individuals compared with individuals treated before seroconversion and individuals treated at a CD4^+^ T cell count <350 cells/μl blood.

To evaluate whether the time of ART initiation had in impact on the HIV-1-specific CD8^+^ T cells, we measured the cytokine responses following stimulation with pools of peptides covering the entire HIV-1 proteome. The HIV-1-specific CD107a, cytokine (IFNγ, IL-2 and TNFα) and chemokine (MIP-1β) responses by CD8^+^ T cells differed between ART-naïve individuals and ART-treated individuals. However, HIV-1-specific responses did not differ between the groups that initiated ART at different stages of disease. Interestingly, individuals with ART initiation before seroconversion were the only group with a five-parameter HIV-1-specific cytokine response that included IL-2 (Fig 3A), although this result did not reach statistical significance (Fig 3B). The overall HIV-1-specific responses (to the five peptide pools of peptides covering the entire HIV-1 proteome, considered collectively) were similar in the three different groups, but ART-naïve individuals had a significantly greater CD107a response compared with treated individuals, independent of when treatment was initiated (Fig 3B). In addition, the co-expression of CD107a and IFNγ was significantly higher in ART-naïve individuals compared with treated individuals (Fig 3B). There were no significant differences in the specificity of the CD8^+^ T cells with regard to the time of treatment initiation. However, notably, the five-parameter response in individuals treated before seroconversion was directed against the Nef pool (data not shown). The HCMV-specific response did not differ between the different groups (data not shown). The preservation of a multifunctional response including IL-2 in individuals treated before seroconversion appeared to be HIV specific.

## Discussion

Immune function is impaired during HIV-1 infection but partially restored during ART [20, 21, 23]. One aspect of this immune dysfunction is CD8^+^ T cell exhaustion [5]. In this study, we investigated whether restoration of CD8^+^ T cell function was dependent on the time of initiation of therapy.

The frequency of exhausted CD8^+^ T cells expressing the inhibitory markers 2B4 and PD-1, particularly when co-expressed or expressed together with CD160, was lower in ART-treated individuals compared with ART-naïve individuals, independent of the time of treatment initiation. The decrease in these markers after treatment initiation has been described before [23], but an analysis in the context of early initiation of ART has not been previously performed. In our analysis, early initiation of ART was defined as ART introduced at a CD4^+^ T cell count >350 cells/μl blood. Phenotype was assessed on whole blood. Phenotype analysis was only available from four individuals treated before seroconversion, the individuals treated before seroconversion and the individuals treated at a CD4^+^ T cell count >350 cells/μl were therefore analyzed as one group. However, individuals treated before seroconversion did not bias the results when combined with individuals treated at a CD4^+^ T cell count >350 cells/μl. All ART-treated individuals included in this study had been virologically suppressed for at least two years. Our results suggest that the time of initiation of ART is important for restoration of CD8^+^ T cell function, as measured based on the expression of inhibitory markers in the total circulating CD8^+^ T cell population; however, more than 2 years of ART was sufficient to accomplish this, and early initiation of therapy was not required. It is possible that a shorter duration of ART would be sufficient to accomplish the same improvement in the CD8^+^ T cell phenotype. For example, we have previously shown that for NK cells, six months of ART is sufficient for a significant restoration of phenotype [24].

The composition of cytokines in HIV-1-specific CD8^+^ T cell responses in individuals with early ART initiation was not significantly different from that in individuals treated at a CD4^+^ T cell count <350 cells/μl blood (median 189, range 155–213). However, all treated individuals deviated from ART-naïve individuals. These results are in line with previous studies describing a decline in the magnitude and breadth of HIV-1-specific CTL responses after initiation of ART [22, 25, 26]. Interestingly, we did observe that individuals treated before seroconversion were the only group capable of responding via all five functional parameters measured here. The response was directed against Nef (though not significant). This finding is in agreement with previous studies reporting that the earliest T cell responses are often specific for Env or Nef [27, 28]. The preservation of HIV-1-specific CD8^+^ T cell function when ART is initiated before seroconversion is in agreement with the cases in which ART was initiated at the time of primary infection and long-term immunological control was achieved after treatment interruption [17, 18]. Here, individuals treated before seroconversion, often initiate treatment as they present with a symptomatic seroconversion, if this could affect the results can only be speculated.

It would be intriguing to investigate the cytotoxic function of CD8^+^ T cells from individuals treated before serconversion in an *ex vivo* viral suppression assay [29] to determine whether HIV-1-specific CD8^+^ T cell cytotoxicity is associated with a broader cytokine profile. Previous studies could not demonstrate an association between the functional profile of Nef-specific CD8^+^ T cell responses and viral load and/or the CD4^+^ T cell count after ART [30]. However, multifunctional cytokine production including IL-2 has been correlated with the HIV-1-specific suppressive function of expanded epitope-specific CD8^+^ T cell lines [31]. An association between *ex vivo* HIV-1 suppression by CD8^+^ T cells and IFNγ responses to Gag has also been demonstrated in HIV-1 controllers [32]. The IFNγ response to Gag was higher in individuals treated before seroconversion (data not shown) than in ART-naïve individuals or individuals treated at a CD4^+^ T cell count <350 cells/μl, but this difference was not statistically significant. If individuals treated before seroconversion have any advantage associated with more cytotoxic CD8^+^ T cells compared with individuals treated at a CD4^+^ T cell count <350 cells/μl blood should be investigated in a future study.

The HIV-1-specific CD8^+^ T cell responses in ART-naïve individuals were dominated by CD107a expression. This finding is in line with our previous work [22], in which CD107a, IFNγ, TNFα and MIP-1β, but not IL-2, dominated the responses including 1–4 functional parameters, demonstrating that a five-parameter response is lost during HIV-infection. Several studies have shown that the HIV-1-specific cytokine responses in long-term non-progressors or slow progressors were associated with more polyfunctional HIV-1-specific T cell responses [33, 34]. To prevent any bias due to long-term or slow progressors, samples from ART-naïve individuals with a viral load <2000 copies/ml plasma were excluded from the intracellular cytokine staining assay in the present study. The viral load in ART-naïve individuals did not seem to bias the CD8^+^ T cell phenotype in the ART-naïve group.

Previous studies have demonstrated an association between the co-expression of inhibitory markers and lower expression of IL-2 and IFNγ by CD8^+^ T cells [13]. The phenotype of the cytokine-producing CD8^+^ T cells was not measured in the present study, but it could be hypothesized that cells from individuals treated before seroconversion would demonstrate a different marker profile compared with CD8^+^ T cells from individuals treated at CD4^+^ T cell count below 350 copies per ml. Phenotype analysis was performed on whole blood because significant differences were found in the frequency of CD8^+^ T cells expressing all of the studied markers when comparing whole blood with PBMCs (data not shown). The deviation between marker expression on PBMCs and CD8^+^ T cells from whole blood as well as the treatment status of the studied individuals could partially explain some of the disparity between our results and previous findings. Finally, the lower number of studied individuals is a limitation of this study.

In conclusion, the total population of circulating CD8^+^ T cells in individuals receiving ART expressed similar levels of the inhibitory markers PD-1, 2B4 and CD160 as total circulating CD8^+^ T cells in HIV-negative individuals did, irrespective of the time of ART initiation. This result suggests that more than 2 years of ART can restore the overall function of CD8^+^ T cells, even if introduced late during the course of disease. However, to preserve multifunctional, HIV-1-specific CD8^+^ T cells, ART might have to be initiated early, before seroconversion. As HIV-1 infection is rarely diagnosed before seroconversion, natural HIV-1-specific CD8^+^ T cells may not be rescued from the immune exhaustion observed during chronic infection. However, a combination of ART and a therapeutic vaccine, with the aim of inducing novel HIV-1-specific CD8^+^ T cell responses, could be a successful approach, regardless of the time of treatment initiation.

## Supporting Information

S1 Fig Detailed gating strategy for assessment of receptor expression on CD8^+^ T cells. The cells were initially gated on a forward-scatter area (FSC-A) versus height (FSC-H) plot to exclude doublets from the analysis. The lymphocytes were identified in a side-scatter area (SSC-A) versus FSC-A plot. The dead cells were confirmed to be V450 bright and were excluded in an SSC-A versus V450 plot. CD8^+^ T cells were identified as CD3^+^CD8^+^. We defined effector CD8^+^ T cells as CD45RO^-^CD27^-^CCR7^-^, naïve cells as CD45RO^+^CD27^+^CCR7^+^, effector memory cells as CD45RO^+^CD27^-^CCR7^-^, central memory cells as CD45RO^+^CD27^+^CCR7^+^ and transitional memory cells as CD45RO^+^CD27^+^CCR7^-^.
(PDF)

S2 Fig Gating strategy for intracellular staining for CD107a and cytokines. This plot is illustrating the response towards the positive control (SEB). The cells were initially gated on a forward-scatter area (FSC-A) versus height (FSC-H) plot to exclude doublets from the analysis. The lymphocytes were identified in a side-scatter area (SSC-A) versus FSC-A plot. The dead cells were confirmed to be V450 bright and were excluded in an SSC-A versus V450 plot. CD3^+^CD4^-^CD8^+^ cells were identified, followed by identification of cells positive for each cytokine and CD107a.
(PDF)

S3 Fig FACS plot of HIV-specific response from an individual representing the group; Individuals treated before seroconversion. (PDF)

S4 Fig FACS plot of HIV-specific response from an individual representing the group; CD4^+^ T cell count >350 cells/μl. (PDF)

S5 Fig FACS plot of HIV-specific response from an individual representing the group; CD4^+^ T cell count <350 cells/xl. (PDF)

S6 Fig FACS plot of HIV-specific response from an individual representing the group; ART naïve. (PDF)
