# Supplementary figures and images for: Initiation of Antiretroviral Therapy (ART) at Different Stages of HIV-1 Disease Is Not Associated with the Proportion of Exhausted CD8+ T Cells
Source: PLoS One. 2015 Oct 1;10(10):e0139573. doi: 10.1371/journal.pone.0139573 (PMC4591005; doi:10.1371/journal.pone.0139573)

# Supporting Information 1

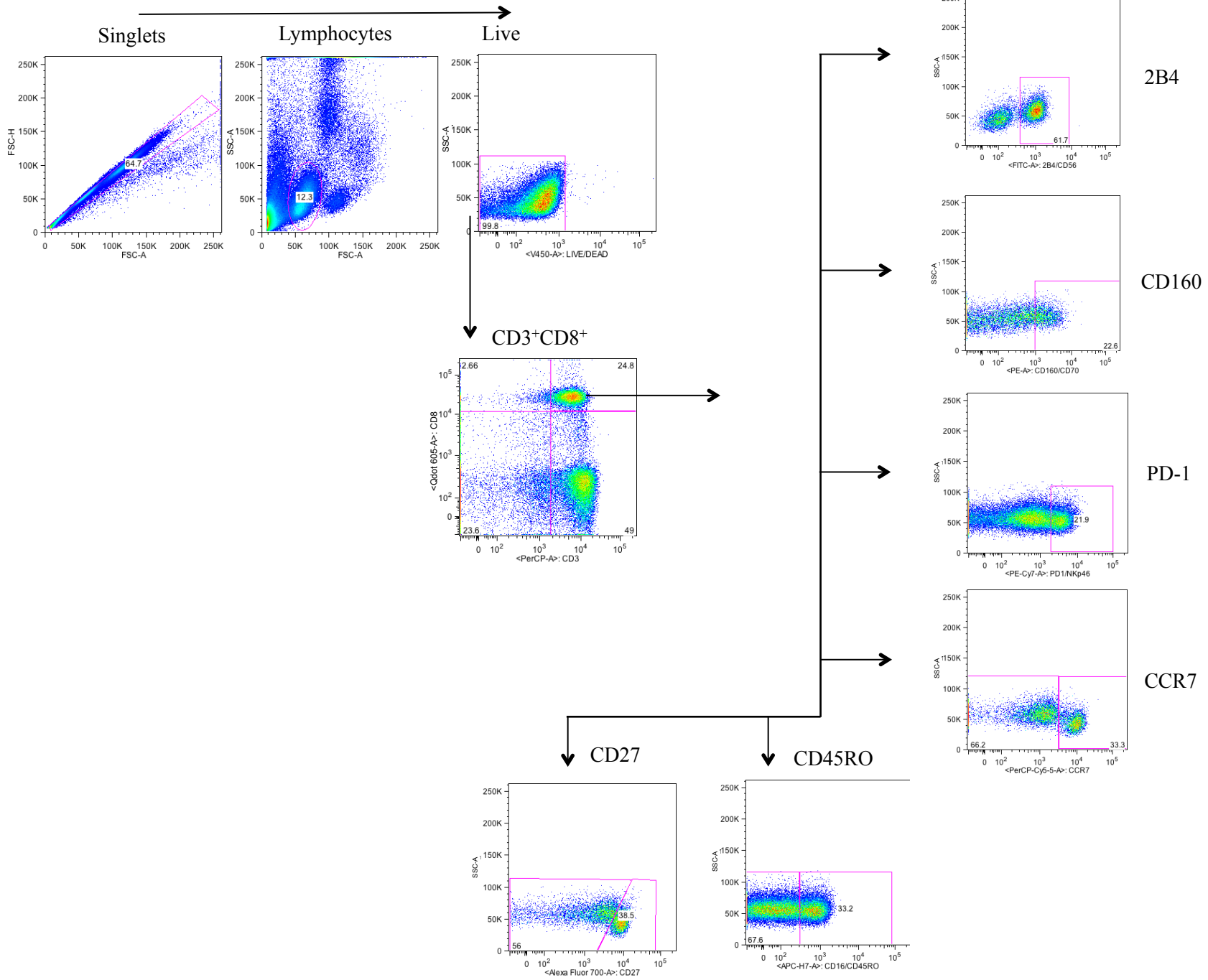

Supplement: S1 Fig — The cells were initially gated on a forward-scatter area (FSC-A) versus height (FSC-H) plot to exclude doublets from the analysis. The lymphocytes were identified in a side-scatter area (SSC-A) versus FSC-A plot. The dead cells were confirmed to be V450 bright and were excluded in an SSC-A versus V450 plot. CD8+ T cells were identified as CD3+CD8+. We defined effector CD8+ T cells as CD45RO-CD27-CCR7-, naïve cells as CD45RO+CD27+CCR7+, effector memory cells as CD45RO+CD27-CCR7-, central memory cells as CD45RO+CD27+CCR7+ and transitional memory cells as CD45RO+CD27+CCR7-. (PDF) [file pone.0139573.s001.pdf]

Supporting Information 2

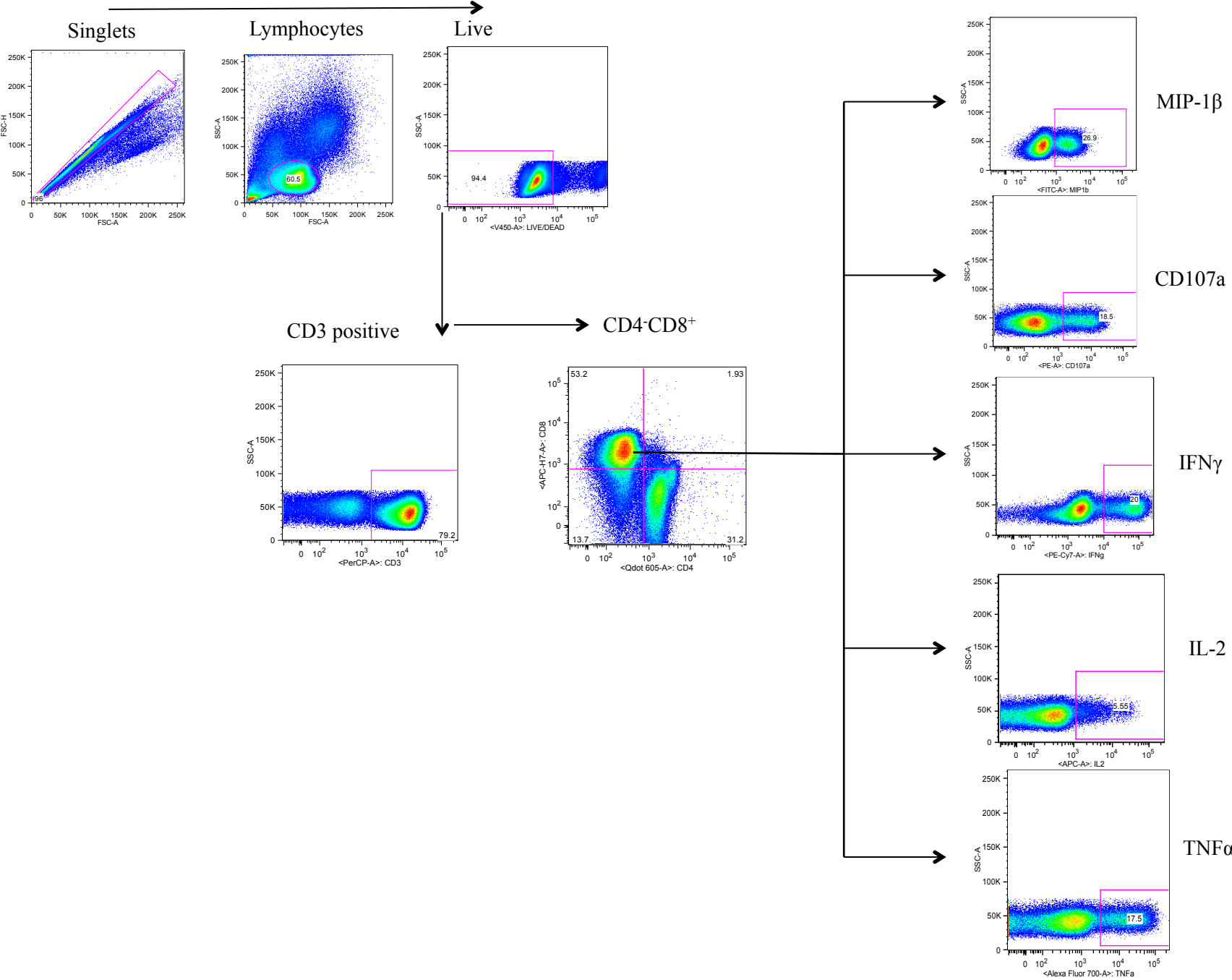

Supplement: S2 Fig — This plot is illustrating the response towards the positive control (SEB). The cells were initially gated on a forward-scatter area (FSC-A) versus height (FSC-H) plot to exclude doublets from the analysis. The lymphocytes were identified in a side-scatter area (SSC-A) versus FSC-A plot. The dead cells were confirmed to be V450 bright and were excluded in an SSC-A versus V450 plot. CD3+CD4-CD8+ cells were identified, followed by identification of cells positive for each cytokine and CD107a. (PDF) [file pone.0139573.s002.pdf]

# Supporting information 3: ART before seroconversion, (nef)

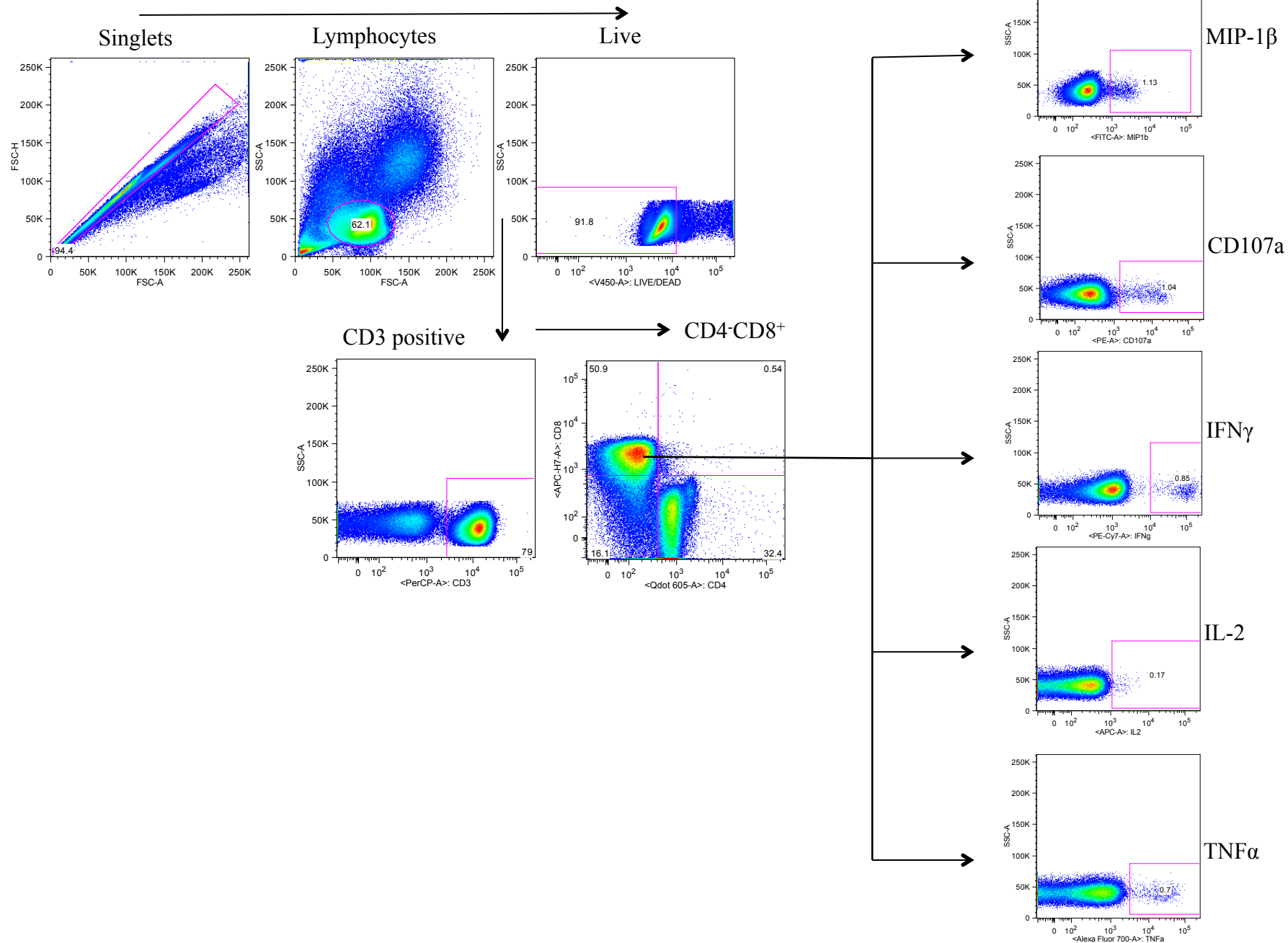

Supplement: S3 Fig — (PDF) [file pone.0139573.s003.pdf]

# Supporting information 4: ART at CD4<sup>+</sup> T cell count >350 cells/ $\mu$ l, (gag)

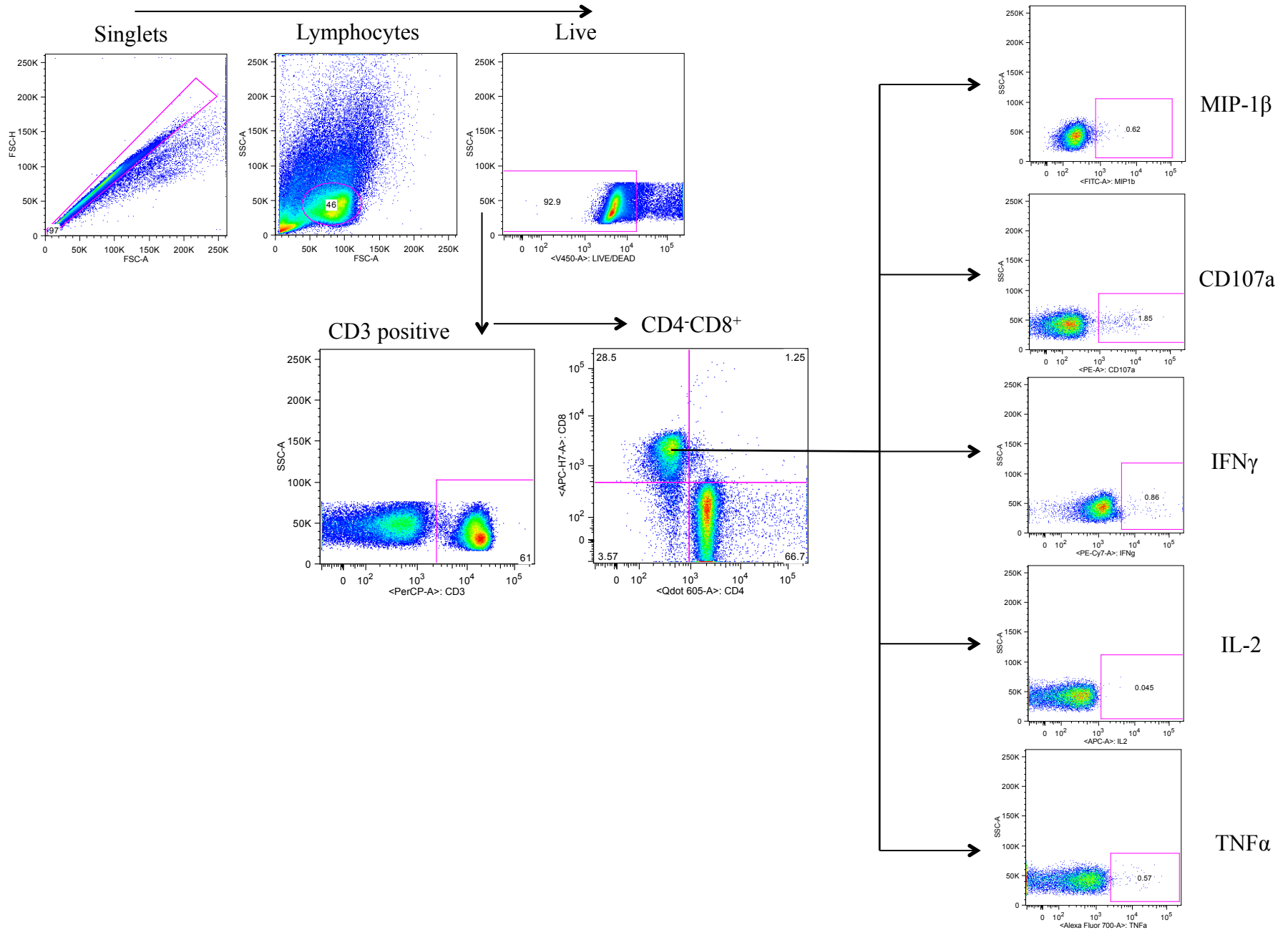

Supplement: S4 Fig — (PDF) [file pone.0139573.s004.pdf]

# Supporting information 5: ART at CD4<sup>+</sup> T cell count <350 cells/ $\mu$ l, (nef)

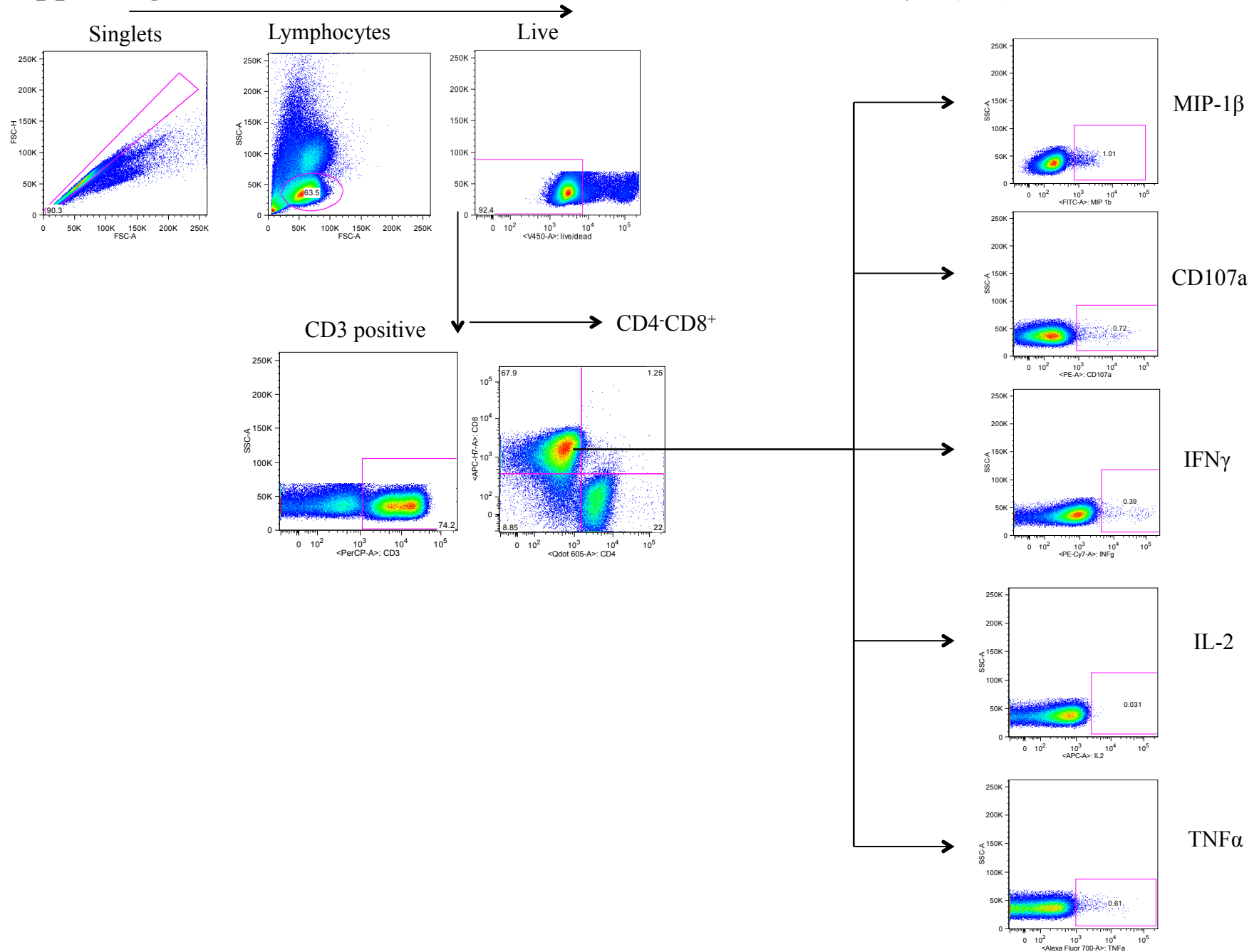

Supplement: S5 Fig — (PDF) [file pone.0139573.s005.pdf]

Supporting information 6: ART-naïve, (nef)

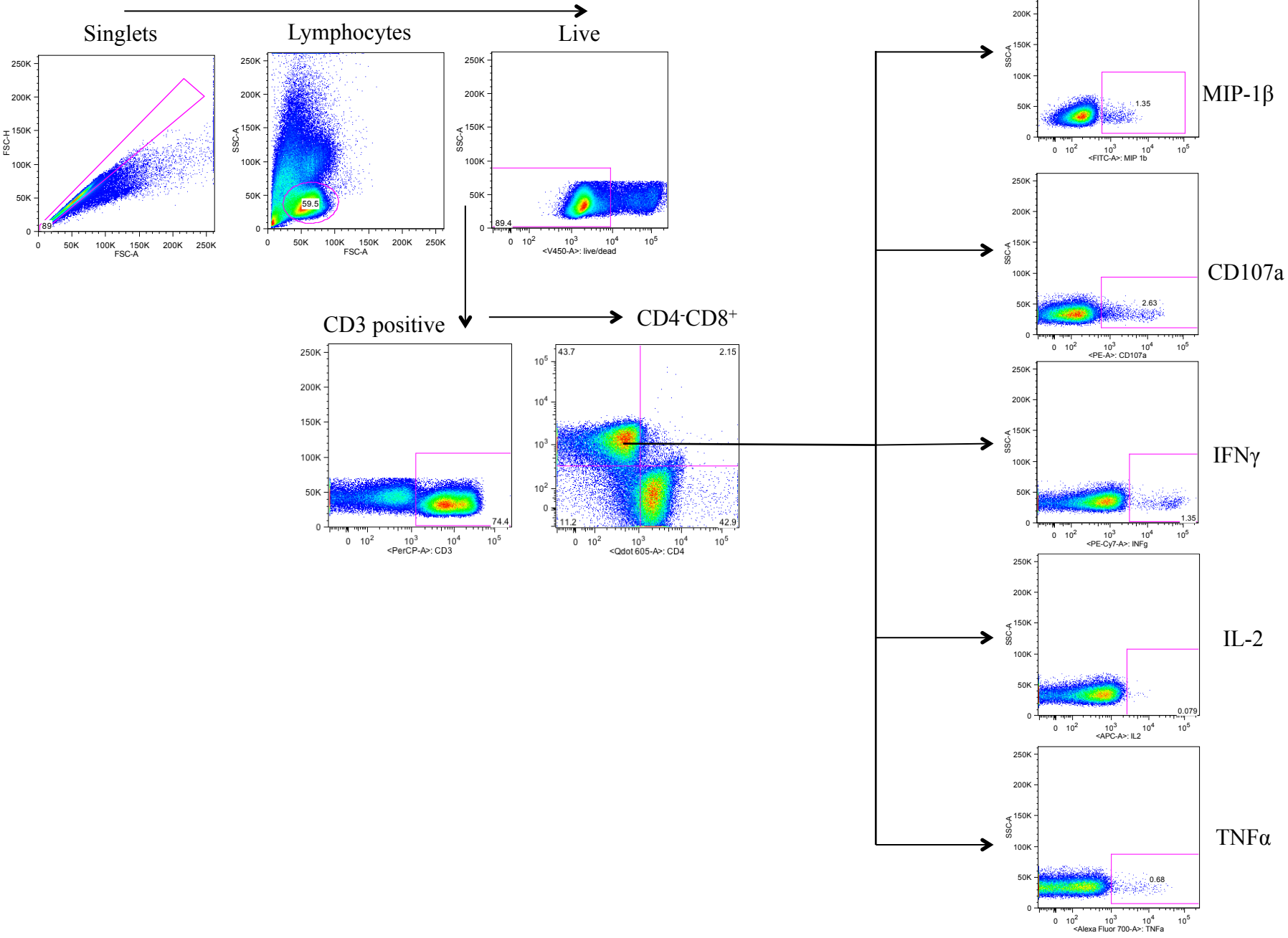

Supplement: S6 Fig — (PDF) [file pone.0139573.s006.pdf]
